# Public health round-up

**DOI:** 10.2471/BLT.15.010215

**Published:** 2015-02-01

**Happy universal health coverage day**

Universal Health Coverage Day was launched on 12 December by a group of health and development organizations to highlight the importance of access to health services for everyone without forcing them into poverty in a web-based campaign.

**Figure:**
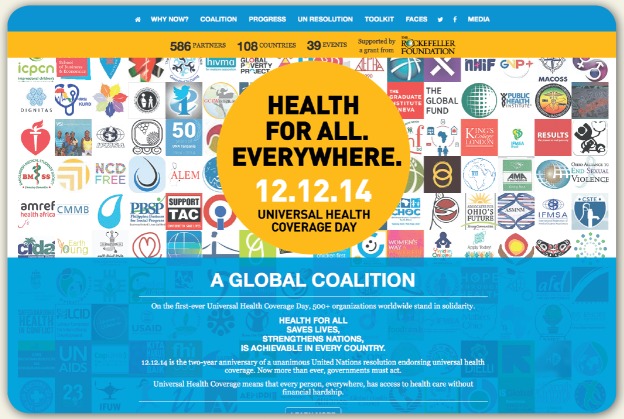
WHO

## Sustainable funding for UHC

Financial bottlenecks – budget shortfalls, irregular funding flows, lack of flexibility in the use of public revenues – are major challenges for countries as they strive towards providing universal health coverage (UHC).

To help countries achieve sustainable funding for UHC, World Health Organization (WHO) economists are developing a package of technical support for health ministries, so that they can work more effectively with the ministries responsible for government finance and planning decisions.

This month, WHO economists will join health and budget officials from the 34 Organisation for Economic Cooperation and Development (OECD) countries to find solutions to the challenges of achieving sustainable funding for their health systems. The meeting will take place on 16 and 17 February.

It will be the fourth annual meeting of the Joint OECD Network on Fiscal Sustainability of Health Systems, which was established in 2011, driven in part by finance ministries’ concerns about the fiscal sustainability of the economy, given the large share of the budget allocated to health. 

“This network facilitates understanding and dialogue by bringing together senior officials from health and budget ministries to identify key challenges and the analytical work that is needed to make progress,” said Joe Kutzin, a health economist from WHO’s department of Health Systems Governance and Financing.

“We’d like to adapt these mechanisms for use in low- and middle-income countries,” he said, adding: “Rolling out universal health coverage requires financial reform in countries, and health ministries cannot do this alone. It requires close collaboration with finance ministries.”

Last December, WHO organized and hosted a meeting, entitled Fiscal Space, Public Finance Management and Health Financing, in Montreux, Switzerland, to look at how to provide sustainable funding for UHC.

Health and finance officials and experts from a dozen countries participated, as did staff of several partner agencies, including the World Bank, the Global Fund to fight AIDS, Tuberculosis and Malaria, PEPFAR and the GAVI Alliance, UNAIDS, the United Kingdom Department for International Development, the Providing for Health Network, and the OECD.

http://www.who.int/health_financing/partner_agencies/event

## Stronger health systems

Representatives from about 30 countries, 12 United Nations agencies, the World Bank, health funders and nongovernmental organizations gathered at WHO headquarters in Geneva from 10 – 11 December to discuss how to strengthen health systems in Guinea, Liberia and Sierra Leone.

The three countries have been ravaged by the worst Ebola virus disease outbreak on record, with 21 373 cases and 8468 deaths as of 13 January this year.

“The answer to stopping Ebola outbreaks of this amplitude is strengthening health systems,” said Dr Marie-Paule Kieny, WHO Assistant-Director General for health systems and innovation. 

“But more importantly than simply strengthening existing capacity for Ebola, countries need to create resilient integrated systems that can be responsive and proactive to any future threat,” she said.

Participants agreed that integrated care – rather than a silo approach to health programmes – is the best approach to strengthening health systems and that universal health coverage, where it has been achieved, has made such integrated approaches practical and affordable.

An essential part of health systems strengthening in these countries should be building and reinforcing strong laboratory capacity and surveillance systems, so that these countries are able to comply with the requirements of the International Health Regulations.

“To achieve this, we must ensure national ownership, local action and full support of development partners,” Kieny said in a commentary published on the WHO website in December.

http://www.who.int/csr/disease/ebola/health-systems

## Reversing the epidemic

Premature deaths from noncommunicable diseases (NCDs) can be significantly reduced through government policies on smoking tobacco, harmful use of alcohol, unhealthy diets and physical inactivity, according to a new WHO report.

Most premature NCD deaths – such as heart and lung diseases, stroke, cancer and diabetes – are preventable. Of the 38 million lives lost to NCDs in 2012, 16 million or 42% were premature ‒ before the age of 70 – and avoidable, according to the *Global status report on noncommunicable diseases 2014*.

Almost three quarters of all NCD deaths (28 million), and 82% of the 16 million premature deaths, occur in low- and middle-income countries.

“The global community has the chance to change the course of the NCD epidemic,” said WHO Director-General Dr Margaret Chan at the launch of the report last month.

“By investing just US$ 1‒3 dollars per person per year, countries can dramatically reduce illness and death from NCDs. In 2015, every country needs to set national targets and implement cost-effective actions. If they do not, millions of lives will continue to be lost too soon.”

The report provides a baseline for monitoring implementation of the *Global action plan for NCDs 2013–2020*, aimed at reducing the number of premature deaths from NCDs by 25% by 2025.

The plan includes nine voluntary global targets that address key NCD risk factors, such as tobacco use, salt intake, physical inactivity, high blood pressure and harmful use of alcohol.

The report lists “best buy” or cost‒effective, high-impact interventions recommended by WHO, such as banning all forms of tobacco advertising, replacing trans fats with polyunsaturated fats and restricting or banning alcohol advertising.

http://www.who.int/chp/ncd_global_status_report/en/

**Cover photo**

A student reads a book in Stone Town, on the Tanzanian island of Zanzibar, where people are benefiting from WHO programmes to strengthen the provision of maternal, newborn and child health, immunization and district health services among others.

**Figure:**
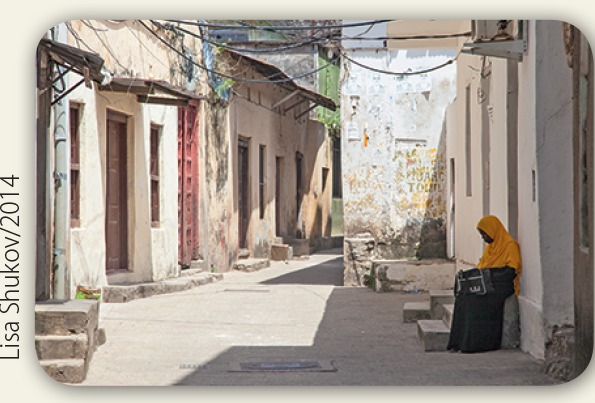
Lisa Shukov/2014

## MenAfriVac® now for infants

A vaccine for meningitis A that was introduced in Africa four years ago for people aged 1–29 years has now been approved by WHO for infants as well.

The WHO decision announced on 9 January means that the new, 5 µg dose of the meningitis A vaccine meets international standards of quality, safety and efficacy and can, therefore, be administered to children younger than one year of age in Africa.

The vaccine, called MenAfriVac®, is manufactured by Serum Institute of India Ltd.

“Initial mass vaccination campaigns with MenAfriVac® have been highly effective in reducing the number of meningitis A cases,” said Dr Marie-Pierre Préziosi, director of the Meningitis Vaccine Project (MVP). The MVP partnership between WHO and nongovernmental organization PATH closed on 31 December last year having achieved its goals.

“But epidemics will return when rising numbers of unprotected newborns become a larger proportion of the total population over time. Now, with this decision, health officials will be able to ensure that population-wide protection is sustained by routinely immunizing infants,” she said.

In the four years since its introduction in Africa, the vaccine has had an immediate and dramatic impact in breaking the cycle of meningitis A epidemics. The approval by WHO, through its prequalification process, for use of the vaccine in infants is essential to prevent such epidemics.

More than 450 million people live within the “meningitis belt”, a band stretching across 25 African countries, from Senegal in the west to Ethiopia in the east. For more than a century, these countries have had devastating outbreaks of the highly contagious disease. 

http://www.meningvax.org

## Ending childhood obesity

Members of a WHO Commission met last month to start work on a draft report setting out the public health interventions that countries can use to end the epidemic of childhood obesity.

Last year WHO Director-General Dr Margaret Chan set up a high-level Commission on Ending Childhood Obesity, comprising 15 experts from diverse backgrounds. It is supported by a 21-member working group on science and evidence and another working group of 24 experts on implementation, monitoring and accountability.

Next month, an online consultation will be held to invite input and comments. Over the course of the year, the commission and working groups will meet again to review the results of the consultation and finalize the report. The final document is due to be delivered to the Director-General by the end of the year.

The global prevalence of overweight in children less than 5 years of age increased from around 5% in 2000 to 6.3% in 2013 and this problem is becoming more prevalent worldwide, especially in Africa and Asia.

http://www.who.int/end-childhood-obesity

**Looking ahead**

**4 February – World Cancer Day.** http://www.who.int/nmh/events/2014/world-cancer-day
**12–14 February – The First World Congress on Ear and Hearing Care, New Delhi, India.** http://www.sh2030worldcong.org/
**14–18 March – 3rd World Conference on Disaster Risk Reduction, Sendai, Japan.** http://www.wcdrr.org/
**20 March – World Oral Health Day.** http://www.worldoralhealthday.com/fdi-launches-its-world-oral-health-day-2015-smile-for-life-campaign/
**22 March – World Water Day.** This year marks the end of the International Decade for Action: Water for Life 2005–2015. http://www.who.int/water_sanitation_health/decade2005_2015
**7 April –** This year **World Health Day** is devoted to the subject of food safety.
http://www.who.int/campaigns/world-health-day/2015/event

